# ^13^C Metabolic Flux Analysis Identifies an Unusual Route for Pyruvate Dissimilation in Mycobacteria which Requires Isocitrate Lyase and Carbon Dioxide Fixation

**DOI:** 10.1371/journal.ppat.1002091

**Published:** 2011-07-21

**Authors:** Dany J. V. Beste, Bhushan Bonde, Nathaniel Hawkins, Jane L. Ward, Michael H. Beale, Stephan Noack, Katharina Nöh, Nicholas J. Kruger, R. George Ratcliffe, Johnjoe McFadden

**Affiliations:** 1 School of Biomedical and Molecular Sciences, University of Surrey, Guildford, United Kingdom; 2 Rothamsted Research, National Centre for Plant and Microbial Metabolomics, Harpenden, Herts, United Kingdom; 3 Forschungszentrum Jülich GmbH, Institute of Bio- and Geosciences 1: Biotechnology 2, Jülich, Germany; 4 Department of Plant Sciences, University of Oxford, Oxford, United Kingdom; Harvard School of Public Health, United States of America

## Abstract

*Mycobacterium tuberculosis* requires the enzyme isocitrate lyase (ICL) for growth and virulence *in vivo*. The demonstration that *M. tuberculosis* also requires ICL for survival during nutrient starvation and has a role during steady state growth in a glycerol limited chemostat indicates a function for this enzyme which extends beyond fat metabolism. As isocitrate lyase is a potential drug target elucidating the role of this enzyme is of importance; however, the role of isocitrate lyase has never been investigated at the level of *in vivo* fluxes. Here we show that deletion of one of the two *icl* genes impairs the replication of *Mycobacterium bovis* BCG at slow growth rate in a carbon limited chemostat. In order to further understand the role of isocitrate lyase in the central metabolism of mycobacteria the effect of growth rate on the *in vivo* fluxes was studied for the first time using ^13^C-metabolic flux analysis (MFA). Tracer experiments were performed with steady state chemostat cultures of BCG or *M. tuberculosis* supplied with ^13^C labeled glycerol or sodium bicarbonate. Through measurements of the ^13^C isotopomer labeling patterns in protein-derived amino acids and enzymatic activity assays we have identified the activity of a novel pathway for pyruvate dissimilation. We named this the GAS pathway because it utilizes the Glyoxylate shunt and Anapleurotic reactions for oxidation of pyruvate, and Succinyl CoA synthetase for the generation of succinyl CoA combined with a very low flux through the succinate – oxaloacetate segment of the tricarboxylic acid cycle. We confirm that *M. tuberculosis* can fix carbon from CO_2_ into biomass. As the human host is abundant in CO_2_ this finding requires further investigation *in vivo* as CO_2_ fixation may provide a point of vulnerability that could be targeted with novel drugs. This study also provides a platform for further studies into the metabolism of *M. tuberculosis* using ^13^C-MFA.

## Author Summary

The role of the enzyme isocitrate lyase (ICL) in the metabolism of *Mycobacterium tuberculosis* has been intensively studied since the demonstration that both of the isocitrate lyase genes encoded by this pathogen, *icl*1 and *icl*2 (although some strains only have *icl*1) are essential for the survival of this pathogen in the host. This finding has been generally interpreted as being due to a shift to the consumption of fats in the host. We previously demonstrated increased ICL activity when the vaccine strain of *M. tuberculosis* (BCG) was growing slowly on carbohydrates, suggesting a more extended role for ICL than just fat metabolism. Here we show that the gene *icl*1 is actually essential for slow growth on glycerol. By feeding BCG and *M. tuberculosis* cells isotopically labeled carbon sources and using a combination of experimental and computational analysis we identified a novel metabolic route for carbohydrate metabolism in which ICL is a key enzyme. We also demonstrated that *M. tuberculosis* is able to acquire biomass carbon from CO_2_. As CO_2_ is abundant in the human host, inhibition of CO_2_ fixation could be exploited in the development of novel drug treatments against tuberculosis.

## Introduction

Initial infection with *Mycobacterium tuberculosis* is either asymptomatic or leads to the development of acute disease (primary tuberculosis). In both cases, infection (including recovery in the absence of treatment) is generally followed by a period of latency which may last for months, years, decades or a lifetime. Reactivated tuberculosis (post-primary) can occur at any time and is a major source of transmission. Unlike many pathogens *M. tuberculosis* does not rely on the production of specific toxins to cause disease. Rather the ability to adapt and survive within the changing and adverse environment provided by the human host during the course of an infection seems to be the secret to the success of *M. tuberculosis*. It is becoming apparent that key to this adaptation is the metabolic reprogramming of *M. tuberculosis* during both the acute and chronic phase of tuberculosis (TB) disease and therefore a more complete understanding of mycobacterial metabolism remains a major goal of TB research.

Our previous studies have investigated the influence of growth rate on metabolism in *M. tuberculosis* [1], [2]. We found significant differences in macromolecular composition between fast and slow-growing cells [1] and demonstrated that the transcriptional response to slow growth *in vitro* has many similarities to the transcriptional response characteristic of the adaptation of *M. tuberculosis* to the macrophage environment [3]. Growth rate modulation is therefore likely to be a significant component of the adaptation of *M. tuberculosis* to the host environment. Yet, the shift to slow growth is much more than a general slowing down of cellular processes. Global mutagenesis analysis demonstrated that growth rate control in *M. tuberculosis* is in fact a carefully orchestrated process which requires a distinct set of genes encoding several virulence determinants, gene regulators, and metabolic enzymes [4]. However, the precise metabolic changes that underlie the shift to slow growth both *in vitro* and *in vivo* remain unclear.

We previously investigated the metabolic response of slow growing glycerol-limited *M. tuberculosis in silico* using flux balance analysis (FBA) of our genome-scale reconstruction of TB metabolism [2]. The most significant result of this study was a predicted increase in flux through the isocitrate lyase reaction during slow growth. This hypothesis was tested experimentally using our chemostat-based experimental system of mycobacterial growth [1]. By directly measuring the activity of isocitrate lyase, in fast and slow growing BCG cells from a chemostat we demonstrated that isocitrate lyase activity was, in accordance with predictions, induced during slow growth [1].

Isocitrate lyase (ICL) has been shown to have an essential role in the survival and persistence of *M. tuberculosis* in both macrophages and mice [5], [6] and is also essential for the persistence of other intracellular pathogens [7], [8]. As this enzyme is apparently absent from human cells, ICL has been intensively investigated as an attractive target for drug development.

The glyoxylate shunt is an anaplerotic pathway important for the metabolism of fatty acids which is mediated by the enzyme ICL along with malate synthase. The essentiality of ICL in the survival and persistence of *M. tuberculosis* has therefore been generally interpreted to be due to this enzyme's role in the glyoxylate shunt and reflect a metabolic shift in the principal carbon source from carbohydrates to fat in the host. However, the ICL's of *M. tuberculosis* also function as methylisocitrate lyases in the methyl citrate cycle where they are involved in the metabolism of propionyl-CoA [9]. More recently ICL has been shown to be essential for intracellular ATP level reduction in a nutrient starvation model of persistence [10] and the glyoxylate shunt has been shown to operate concurrently with an oxidative (tricarboxylic acid) TCA cycle which is completed by an anaerobic α-ketoglutarate ferredoxin oxidoreductase (KOR) [11]. Our *in vitro* enzyme activity data demonstrates a role for ICL during slow growth rate on glycerol [2], a substrate that would be expected to be catabolised via glycolysis and the TCA cycle and provided a first clue that the glyoxylate shunt in *M. tuberculosis* operates under more general conditions.

However, FBA cannot provide unique solutions for intracellular fluxes because of the existence of multiple parallel pathways; so the method could not identify the metabolic pathways utilized by *M. tuberculosis* during slow growth. A more direct means of establishing the intracellular fluxes is through ^13^C metabolic flux analysis (^13^C-MFA). This powerful technique has been successfully applied to identify functional flux states in various microbes [12], [13] and has enormous potential for studying the metabolism of *M. tuberculosis*. The method utilizes the fact that amino acids are constructed from metabolic precursors in central metabolism. Growth of a cell with ^13^C-labeled substrate therefore leads to a pattern of ^13^C incorporation in the proteinogenic amino acids that is determined by the intracellular fluxes to these compounds. Computational tools may then be used to find the values of the intracellular fluxes that are consistent with the pattern of amino acid labeling, as determined by NMR or mass spectrometry.

In this study we further examine the hypothesis that ICL plays a role in dissimilation of carbohydrates in *M. tuberculosis* at slow growth rates using mutational, enzymatic and macromolecular analysis. We demonstrate that ICL is actually essential in slowly growing *Mycobacterium bovis* BCG in a glycerol-limited chemostat. In addition we use ^13^C-MFA to determine the metabolic phenotype of *M. bovis* BCG at fast and slow growth rates and *M. tuberculosis* H37Rv at slow growth rate, in order to provide a system-level understanding of the metabolism of slowly growing mycobacteria and particularly the role of ICL. This approach provides a novel insight into the *M. tuberculosis* metabolism that may have implications for how the tubercle bacillus survives *in vivo*.

## Results

### Deletion of *icl*1 from *M. bovis* BCG

To further explore the role of ICL during slow growth, the *icl*1 gene was deleted from BCG to generate BCGΔ*icl*1 and this was confirmed by Southern blot (Figure S1). Enzyme activity of the resulting mutant (1.32 nmol min^−1^ mg protein^−1^) was less than one tenth that of the wild type value (21.20 nmol min^−1^ mg protein^−1^). The residual activity demonstrates that another gene encodes an active ICL in *M. bovis* BCG. Two ICL genes have been identified in *M. tuberculosis* H37Rv (*icl*1 and *icl*2) but only one (*icl*1) encodes a functional enzyme in this strain [14]. In *M. tuberculosis* CSU93 [14], *M. bovis* [15] and *M. avium* both genes encode active enzymes. The arrangement of the isocitrate lyase genes in the *M. bovis* BCG genome sequence suggests that both enzymes are also functional in this strain and the data presented here confirms this.

### Growth of Δ*icl*1 in continuous culture in a chemostat

**Figure 1:**
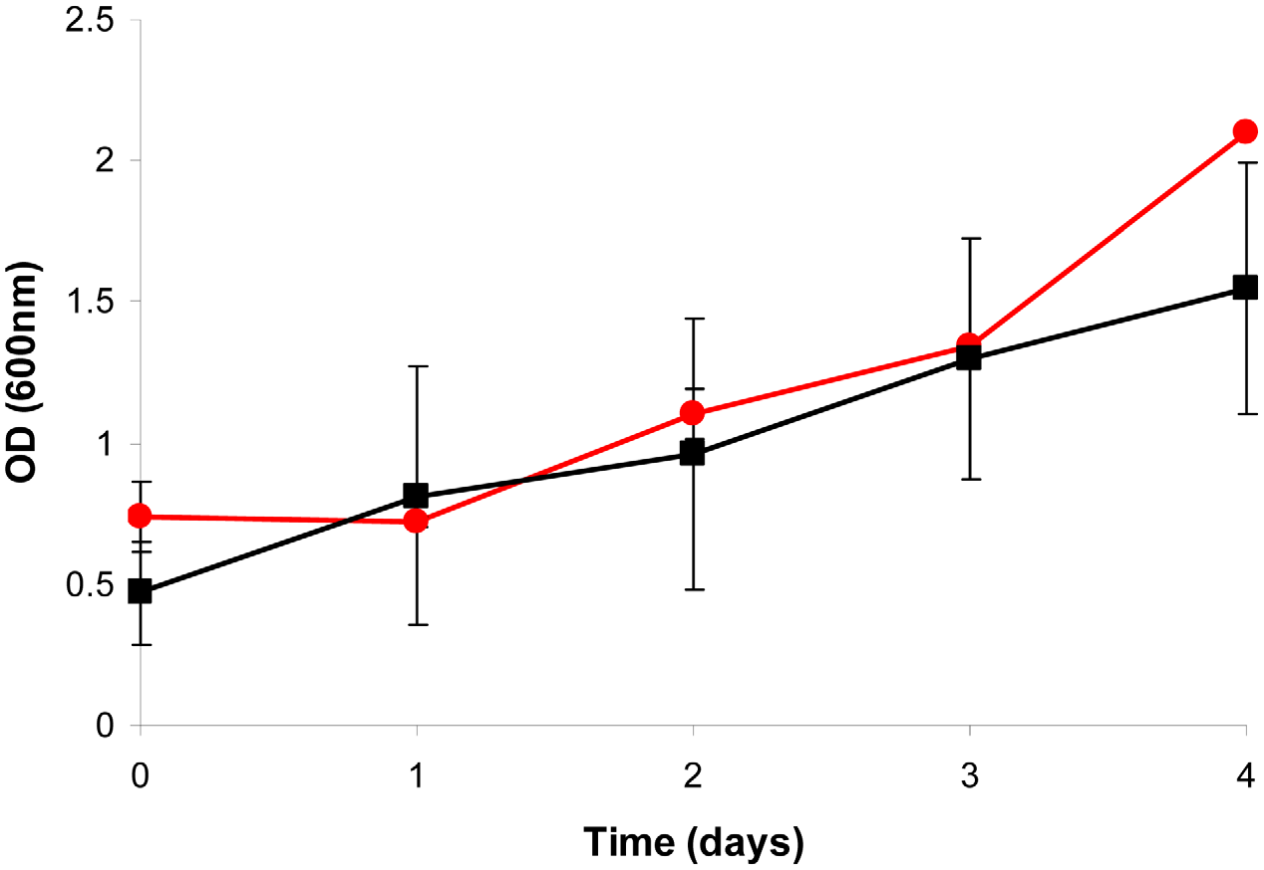
Batch cultivation of *M. bovis* BCG and ▵*icl*1 in a bioreactor. A 10% late log phase culture of *M. bovis* BCG (black line) or ▵*icl*1 (red line) was inoculated into the bioreactor and optical density at 600 nm was monitored for 4 d prior to switching to chemostat mode. Average values and standard deviations are shown from two independent experiments.

**Figure 2:**
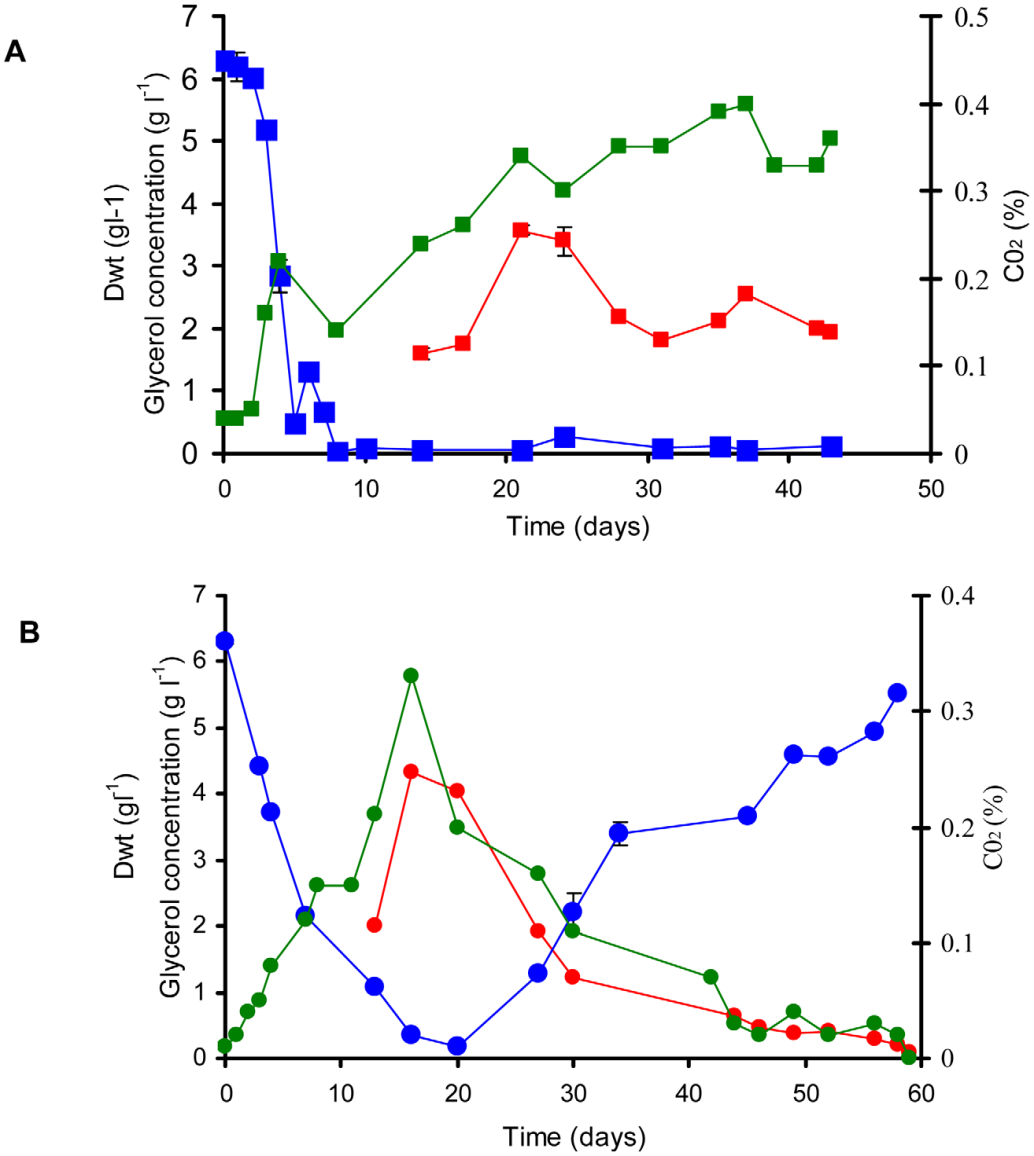
Continuous culture of *M. bovis* BCG and ▵*icl*1 at a dilution rate of 0.01 h^−1^ (t_d_ = 69.3 h). A 10% late log phase culture of *M. bovis* BCG (A) and ▵*icl*1 (B) was inoculated into the bioreactor. The cultures were operated as batch until day 4. Continuous culture was then started at a dilution rate of 0.01 h^−1^ Samples were removed and assayed for biomass (red line). CO_2_ production in the waste gas was measured using a dual tandem sensor gas analyser (green line) and filtered supernatants were assayed for glycerol (blue line). The results are representative of two experiments.

The phenotype of the Δ*icl*1 mutant strain was characterized at two specific growth rates in the chemostat: *µ_S_* = 0.03 h^−1^ (t_d_ = 23 h) and *µ_F_* = 0.01 h^−1^ (t_d_ = 69 h). The physiological [1] and transcriptomic [3] profile and also the genetic requirements [4] of mycobacterial cells at these growth rates have been described previously. The loss of the *icl*1 gene did not impair the ability of the mutant to adapt to growth in the bioreactor, as growth of the mutant strain was indistinguishable from the wild type during the batch phase (Figure 1). However, the wild type and mutant strain behaved very differently after media flow from the feed reservoir into the bioreactor was initiated in order to establish carbon-limited continuous culture conditions. At a dilution rate of 0.01 h^−1^ (corresponding to a doubling time of 69 h), wild type BCG reached a steady state after approximately six volume changes (day 40) after which little variation was observed in the CO_2_ and biomass production rates, indicating that steady state (continuous culture) conditions had been attained (Figure 2). Cells were harvested for analysis at day 42. In contrast, Δ*icl*1 mutant strain appeared unable to establish a steady-state at this growth rate. Instead, the biomass and CO_2_ production of Δ*icl*1 mutant culture declined continuously from day 16 until day 59 when the strain was completely washed out of the chemostat and the experiment was terminated (Figure 2). Consumption of glycerol also differed between the two strains during the course of the experiment (Figure 2). The Δ*icl*1 mutant consumed glycerol much more slowly than the wild type BCG. Growth of the Δ*icl*1 mutant was similar to the wild type strain up to the point at which glycerol became limited, which occurred at day 16 for Δ*icl*1 and at day 10 for the wild type strain (Figure 2). After this point the growth of the wild type adjusted to the rate of nutrient supplied by the chemostat to reach steady state whereas the Δ*icl*1 mutant started to flush out from the bioreactor vessel. These results indicated that loss of *icl*1 significantly changed the growth properties of *M. bovis* BCG even when growing with glycerol as the principal carbon source.

**Figure 3:**
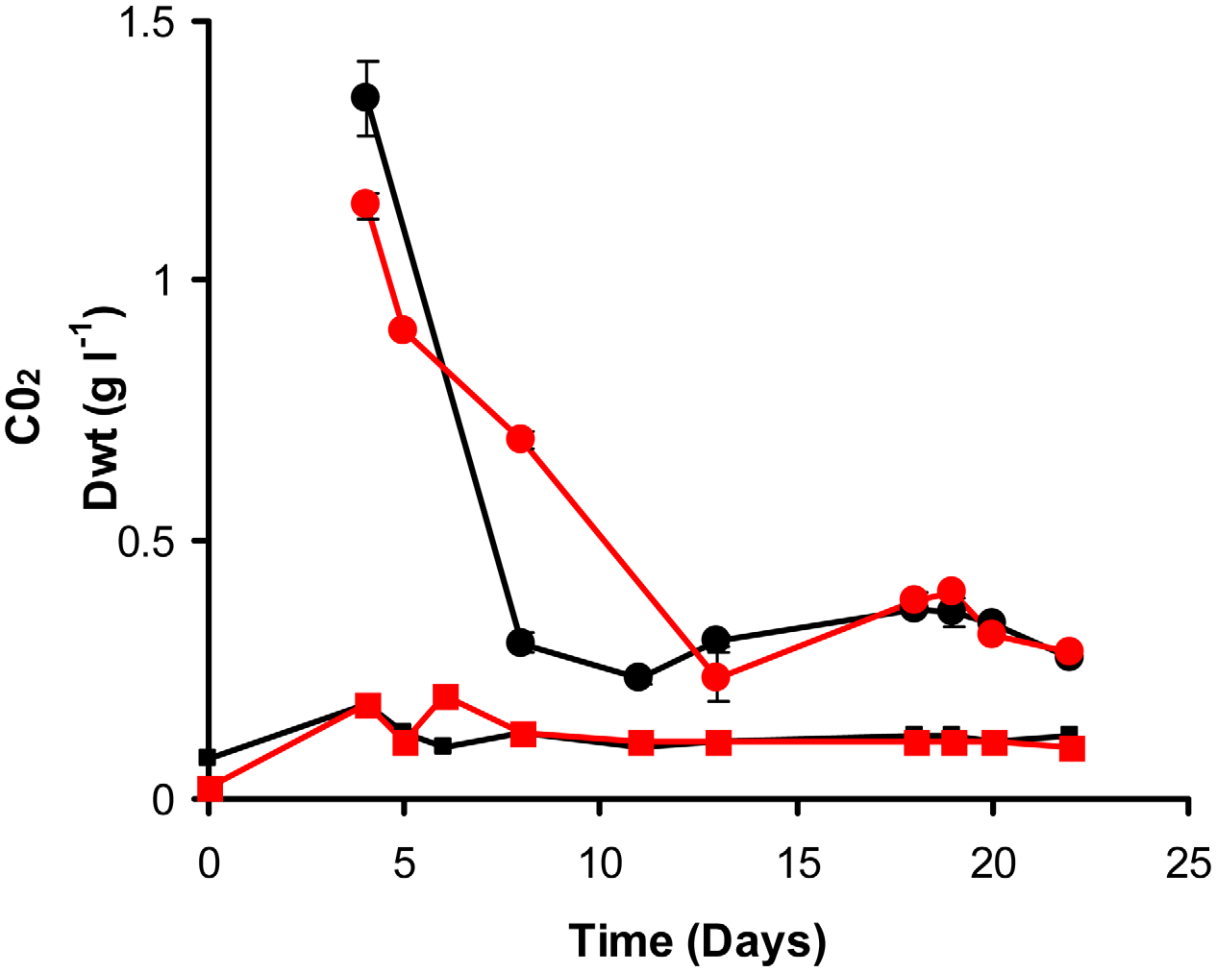
Continuous culture of *M. bovis* BCG and ▵*icl*1 at a dilution rate of 0.03 h^−1^ (t_d_ = 23.1 h). A 10% late log phase culture of *M. bovis* BCG (black line) or ▵*icl*1 (red line) was inoculated into the bioreactor. The cultures were operated as batch until day 4. Continuous culture was then started at a dilution rate of 0.03 h^−1^. Samples were removed and assayed for biomass (circles). CO_2_ production in the waste gas was measured using a dual tandem sensor gas analyser (squares). The results are representative of two experiments.

In contrast, the behavior of the wild type and Δ*icl*1 mutant was similar in the chemostat at a higher dilution rate of 0.03 h^−1^ (corresponding to a doubling time of 23 h) (Figure 3) in that both wild type and mutant attained a steady state after 22 days of growth (five volume changes). There were however significant physiological differences between wildtype and mutant strains (Table 1). A significant decrease in the specific glycerol uptake rate, increased CO_2_ production and an increased biomass yield was observed for the Δ*icl*1 mutant compared with the wild type. Interestingly the Tween 80 consumption rates did not differ significantly between the wild type and mutant strains (Table 1). Macromolecular analysis demonstrated that whereas the relative fraction of lipid was similar for wild type *icl*1 mutant strains (Table 1) the carbohydrate content was markedly decreased in the *icl*1 mutant and this was balanced by an increase in protein content.

**Table 1:** Steady state characteristics of wild type and ▵*icl* strains of *M.bovis* BCG (BCG) and wild type *M. tuberculosis* (MTB) grown in a glycerol-limited chemostat.

Organism	BCG			MTB
Strain	Wild type		▵*icl*	Wild type
Dilution rate (h^−1^)	0.01	0.03	0.03	0.01
Biomass (g dry weight l^−1^)	1.40	0.70	0.30	1.28
CFU (×10^6^ ml^−1^)	164	51.0	26.85	144
Specific glycerol consumption rate (mmol g biomass^−1^ h^−1^)	0.39	0.74	0.57	0.39
Specific Tween 80 consumption rate (mmol g biomass^−1^ h^−1^)	0.002	0.09	0.07	0.003
Specific CO_2_ production rate (mmol g biomass^−1^ h^−1^)	0.57	0.45	0.69	0.23
Protein content (g l^−1^)	22.91*	21.40*	32.74	-
Carbohydrate content (g l^−1^)	29.20*	21.91*	14.37	-
Lipid content (g l^−1^)	33.90*	44.82*	48.14	-
Yield (g bacteria g glycerol^−1^)	0.30	0.46	0.56	0.22

Values are the averages from duplicate chemostat cultures and three independent measurements. * Data from Beste *et al*. (2005).

### Enzyme activities

We have previously demonstrated that the *in vitro* activities of ICL were two-fold higher in slow (t_d_ = 69 h) growing chemostat cultures than in the more rapidly (t_d_ = 23 h) growing cultures [2]. In order to further explore the unexpected role of ICL at slow growth we measured the *in vitro* activities of the competing enzyme, isocitrate dehydrogenase (IDH), and also enzymes involved in downstream anaplerotic reactions (Table 2) in the wild-type strain. We also measured the activity of glycine dehydrogenase (GDH) as this enzyme was shown to act in concert with ICL in Wayne's model of non-replicating persistence [16]. As these enzymes play important roles in controlling the distribution of metabolic flux in the cell this could provide information about the metabolic wiring during slow growth rate. Consistent with elevation of the glyoxylate pathway, *in vitro* activity of IDH was 50% lower at the slow as compared with the fast growth rate (Table 2). The pattern of the anaplerotic phosphoenolpyruvate carboxykinase (PCK) enzyme activity paralleled that of ICL and higher activities were observed at slow growth rate as compared with fast growth rate. The *in vitro* activity of the competing anaplerotic enzyme, pyruvate carboxylase (PCA) was however below the detection level of the assay at both growth rates indicating that this enzyme is either inactive in the growth conditions tested or that the assay conditions were not optimum. Very low levels of malic enzyme (MEZ) were also detected at both growth rates. This is in accordance with previous observations [17]. GDH activity was detected in almost equal amounts at both growth rates.

**Table 2:** *In vitro* enzyme activities in crude cell extracts of glycerol limited chemostat cultures of *Mycobacterium bovis* BCG.

Dilution rate (h^−1^)	0.01	0.03
Doubling time (h)	69.3	23.1
Enzyme activities (nmol mg protein^−1^ min^−1^)		
Isocitrate lyase *	42.67±2.68	21.20±0.69
Isocitrate dehydrogenase	31.6±15.9	66.8±11.9
PEP carboxykinase	52.42±3.88	36.71±1.78
Pyruvate carboxylase	<1	<1
Malic enzyme	3.20±0.60	3.36±0.39
Glycine dehydrogenase	7.13±0.227	6.09±0.18

Results represent the average values with standard deviations from duplicate independent chemostat runs. * Data from Beste *et al*. (2007).

### ^13^C MFA of *M. bovis* BCG

In order to complement the enzyme activity data and further explore the metabolic phenotype of slowly growing mycobacterial cells we performed metabolic flux analysis (MFA) using [^13^C_3_]glycerol. The labeling experiments with *M. bovis* BCG were carried out at both fast (t_d_ = 23 h) and slow (t_d_ = 69 h) growth rates. The physiological characteristics of cells at these growth rates are summarized in Table 1. Our initial data demonstrated that, in addition to glycerol, Tween 80 was also utilized by BCG as a carbon source during the chemostat experiments. We therefore developed and utilized an assay to measure Tween 80 uptake (Methods) in the chemostat, and Tween 80 was also included as a carbon source in the metabolic flux analysis. Perhaps surprisingly, significantly less of the available Tween 80 was consumed during slow growth (16%) than at the faster growth rate (43%).

Steady state chemostat cultures were switched to medium containing 20% [^13^C_3_]glycerol and samples were removed for analysis every volume change for a minimum of five volume changes. Labeling of proteinogenic amino acids was measured using gas chromatography coupled to mass spectrometry (GC-MS). The labeling pattern of most of the fragments changed very little between the third and fourth volume change indicating that the culture had reached an isotopic steady state (Table S1). After correction for the naturally occurring isotopes of atoms other than carbon, experimental GC-MS data was compared to data simulated from flux distributions using the 13CFlux software package (Table S3; Table S4) [18].

**Figure 4:**
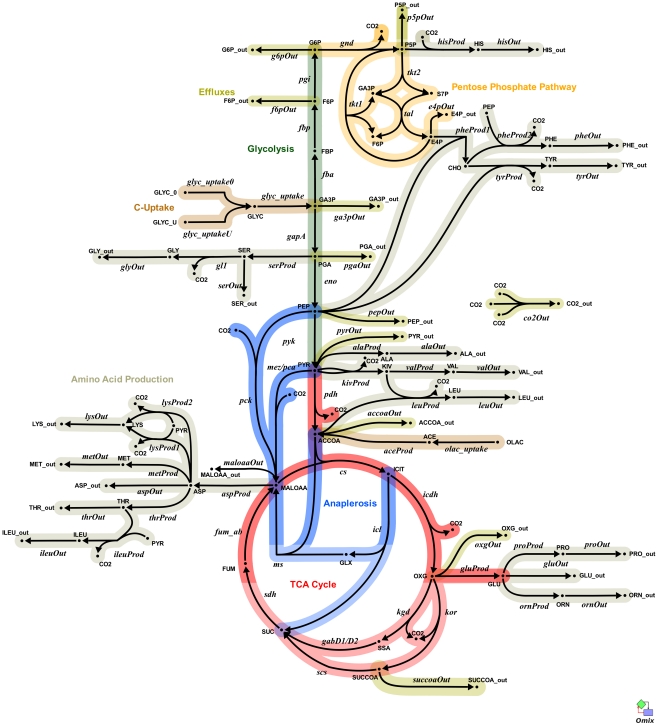
Metabolic network of the central metabolism of *Mycobacterium bovis* BCG. Glycolysis/gluconeogenesis (green), oxidative pentose phosphate pathway (orange), tricarboxylic acid cycle (TCA, pink), anaplerotic reactions (blue), and biosynthesis (grey). Standard abbreviations are used for the amino acids. Metabolite abbreviations: ACCOA, acetyl-CoA; ACE, acetate; CHO, chorismate; E4P, d-erythrose 4-phosphate; F6P, d-fructose 6-phosphate; FBP, d-fructose 1,6-bisphosphate; FUM, fumarate; G6P, d-glucose 6-phosphate; GA3P, glyceraldehyde 3-phosphate; GLX, glyoxylate; GLYC, glycerol; ICIT, isocitrate/citrate; KIV, 2-oxoisovalerate; MALOAA, l-malate-oxaloacetate; OXG, 2-oxoglutarate; R5P,α- d -ribose 5-phosphate/l -ribulose 5-phosphate/l-xylulose 5-phosphate; PEP, phosphoenolpyruvate; PGA, 2-phospho- d -glycerate/3-phospho- d-glycerate; PYR, pyruvate; S7P, sedoheptulose 7-phosphate; SUC, succinate; SUCCOA, succinyl-CoA. Enzyme abbreviations: CS: citrate synthase; ENO: enolase; FBA: fructose-bisphosphate adolase; FBP: fructose-bisphosphatase; FUM:fumurase; GAPA: glyceraldehyde 3-phosphate dehydrogenase; GND: 6-phosphogluconate dehydrogenase; ICL: isocitrate lyase; ICDH: isocitrate dehydrogenase NADP-dependent; KOR/KGD: α-ketoglutarate ferredoxin oxidoreductase/α-ketoglutarate decarboxylase;MEZ/PCA: malic enzyme/pyruvate carboxylase: MDH: malate dehydrogenase; MS: malate synthase; PCK: phosphoenolpyruvate carboxykinase; PDH: pyruvate dehydrogenase; PGI: glucose phosphate isomerase; PYK: pyruvate kinase;SDH: succinate dehydrogenase; SCS: Succinyl CoA synthetase; TAL: transaldolase; TKT1/2: transketolase. Explicit names are also given in Table S6. The picture was generated with Omix, an editor for biochemical networks and visualization tool [http://www.13cflux.net/omix].

For flux calculations we constructed an isotopomer model composed of reactions extracted from our GSMN-TB model [2] and included the reactions of glycolysis, the pentose phosphate pathway, the TCA cycle and anaplerosis. This isotopomer model is applicable to both *M. bovis* BCG and *M. tuberculosis* as current knowledge indicates that these pathways are identical in these two species. The model included fully formulated amino acid biosynthesis pathways and reactions for output of metabolic precursors to biomass, allowing estimate of the fluxes to biomass from each precursor, which are shown as output fluxes in Figure 4 and Table S2. This was a relatively large network for analysis and therefore we simplified this initial network to the point where it could be solvable using the GC-MS data. *M. tuberculosis* and *M. bovis* BCG are peculiar in that two distinct and unconventional TCA cycles are utilized [11], [19]. Lacking the traditional TCA ketoglutarate dehydrogenase (KGD) both *M. tuberculosis* and *M. bovi*s BCG express an anaerobic α-ketoglutarate ferredoxin oxidoreductase (KOR) normally associated with the reductive TCA cycle and this is thought to complete the oxidative TCA cycle [11]. In addition a novel TCA cycle which uses an alternative pathway from α-ketoglutarate to succinate via succinic semialdehyde has also been proposed in *M. tuberculosis* [19]. The existence of this pathway is now called into question due to the re-characterisation of the proposed α-ketoglutarate decarboxylase (KGD) as a 2-hydroxy-3-oxoadipate synthase [20]. However, these pathways cannot be distinguished by ^13^C-MFA as the carbon flow from α-ketoglutarate to succinate is the same irrespective of which route is taken. Consequently both routes are combined and lumped into one flux model. Simplifications were also introduced where the accuracy of flux estimates was predicted to be poor. For example based on available measurements it was also not possible to discriminate between the MEZ and PCA reactions with high confidence so the summed flux is shown between pyruvate and malate/oxaloacetate. The refined network is illustrated in Figure 4 and the component steps defined in Table S2.

In addition, in order to directly compare the fast (D = 0.03 h^−1^, t_d_ = 23 h) and slow (D = 0.01 h^−1^, t_d_ = 69 h) growth rates and also to incorporate information from the enzymatic data the final isotopomer model consists of two sub-models, representing fast and slow growth rates. Both sub-models are structurally identical and are coupled via inequality constraints to reflect the tendencies of the measured *in vitro* enzyme activities (Table 2). 
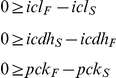
This allowed the parameter estimation process to fit simultaneously all of the measured data for both growth rates without hard coding enzyme activity measurements.

**Figure 5:**
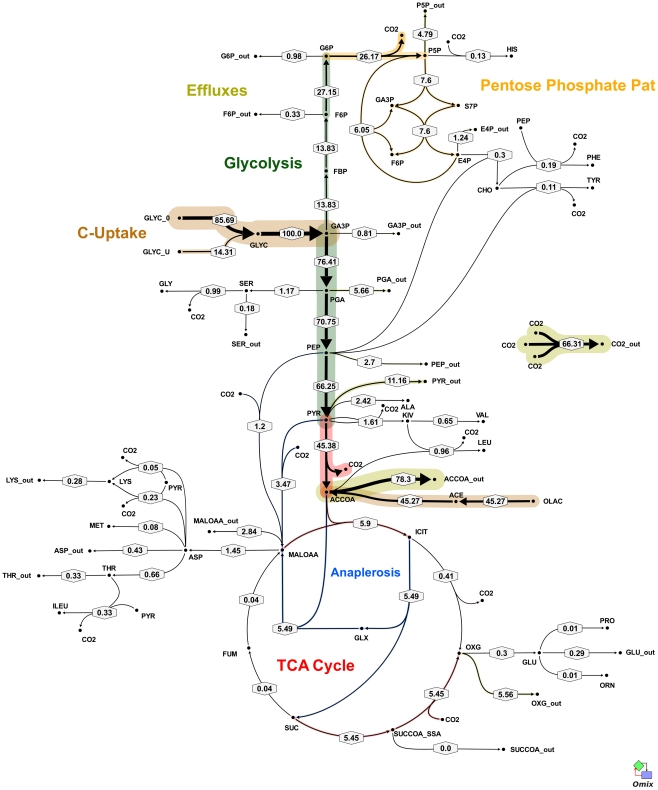
*In vivo* flux distribution of *M. bovis* BCG in glycerol limited continuous culture at fast growth rate (t_d_ = 23 h). Flux values were normalized to the specific glycerol uptake rate which was arbitrarily given the value of 100. Arrows are pointing in the net flux direction and the width of each line is proportional to the underlying flux value. Numerical values of estimated fluxes (Table S5) are indicated on each flux arrow. The abbreviations are as in Figure 4.

**Figure 6:**
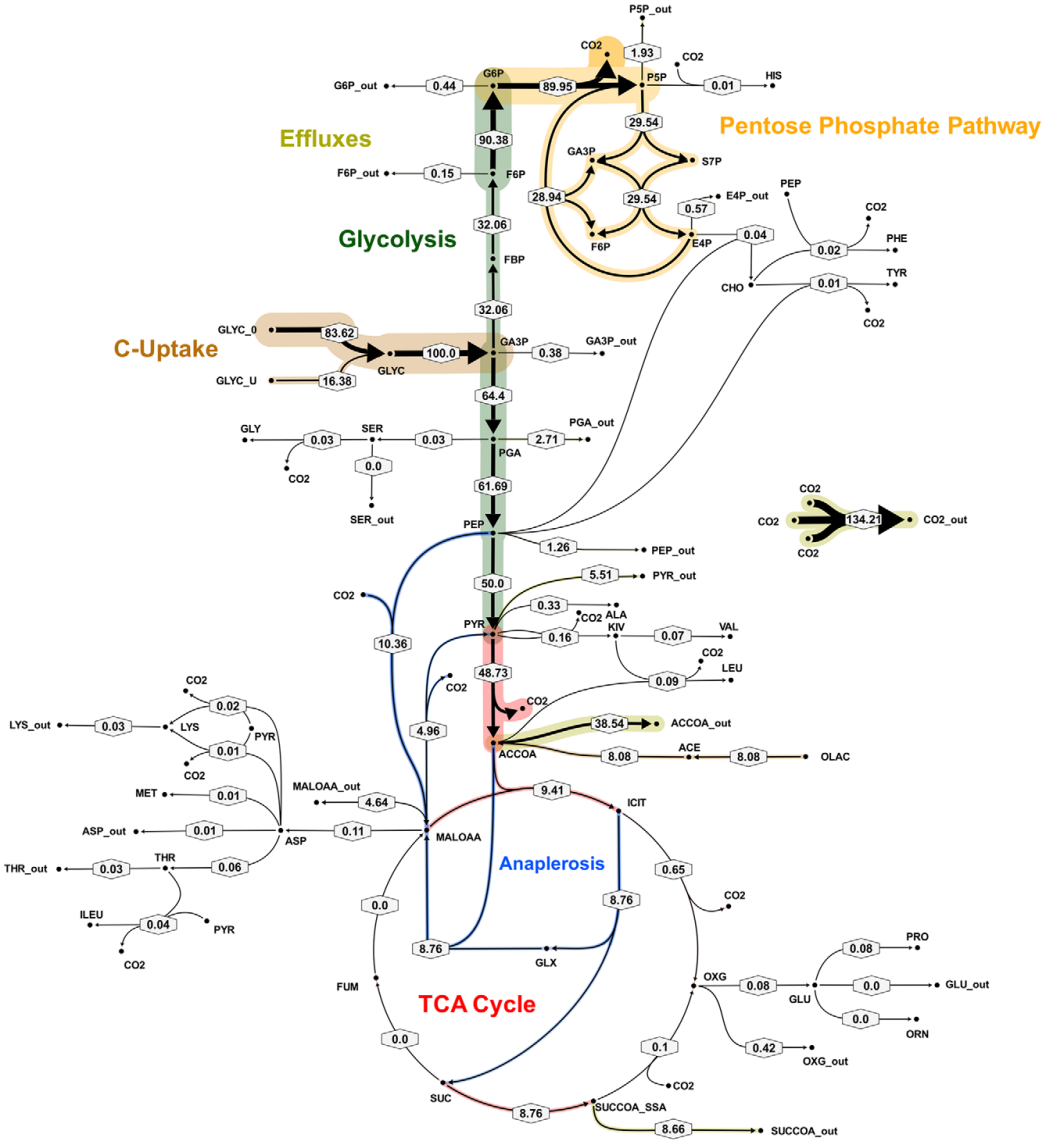
Metabolic flux map corresponding to solution A for *M. bovis* BCG in glycerol limited continuous culture at slow growth rate (t_d_ = 69 h). Flux values were normalized to the specific glycerol uptake rate which was arbitrarily given the value of 100. Mass spectral data of proteinogenic amino acids from steady state chemostat cultures grown on a mixture of 20% [U-13C] glycerol and 80% unlabeled glycerol and the physiological data of Table 1 were used to generate the two alternative flux estimations. Arrows are pointing in the net flux direction and the width of each line is proportional to the underlying flux value. Numerical values of estimated fluxes (Table S5) are indicated on each flux arrow. The abbreviations are as in Figure 4.

**Figure 7:**
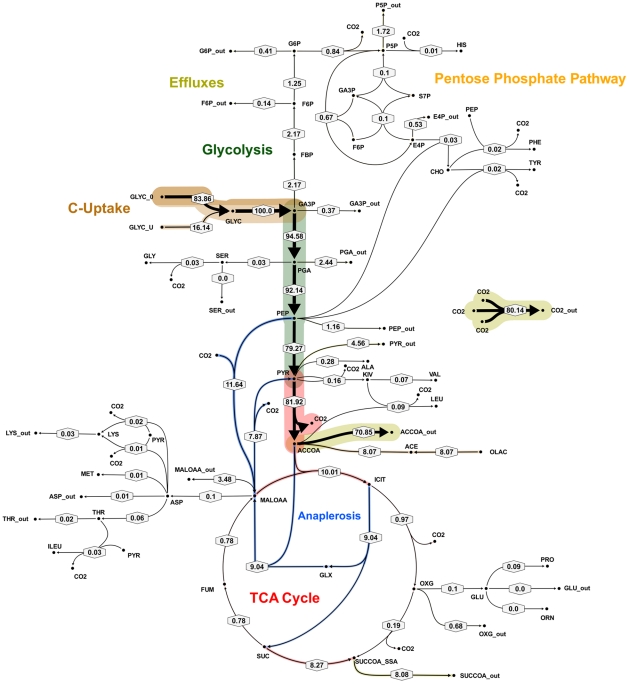
Metabolic flux map B for *M. bovis* BCG in glycerol limited continuous culture at slow growth rate (t_d_ = 69 h). Flux values were normalized to the specific glycerol uptake rate which was arbitrarily given the value of 100. Mass spectral data of proteinogenic amino acids from steady state chemostat cultures grown on a mixture of 20% [U-13C] glycerol and 80% unlabeled glycerol and the physiological data of Table 1 were used to generate the two alternative flux estimations. Arrows are pointing in the net flux direction and the width of each line is proportional to the underlying flux value. Numerical values of estimated fluxes (Table S5) are indicated on each flux arrow. The abbreviations are as in Figure 4.

All fluxes were normalized relative to the rate of glycerol uptake which was considered to be 100. A unique best fit was obtained for the fast growth rate BCG (Figure 5) whereas two significantly different, but equally likely solutions (A and B) were obtained for the slow growth rate (Figure 6 and 7; Table S5). For the slow growth rate therefore it can be concluded that the flux values cannot be determined unambiguously from the available labeling data. This is a relatively common outcome in ^13^C-MFA. The major differences in these two flux solutions are in the operation of the gluconeogenesis, the pentose phosphate pathway and the output of acetyl CoA. However, whilst there are differences between the two slow growth rate solutions they share a common metabolic route for pyruvate dissimilation via the TCA cycle, glyoxylate shunt and anaplerotic reactions and we therefore focus our conclusions on the operation of these pathways.

According to the weak constraints applied to the model (see above) ICL flux was higher at the slow growth rate (8.76–9.04%) than at the fast growth rate (5.49%) for both slow growth rate solutions. In addition, at either the slow or fast growth rate use of the glyoxylate shunt was coupled with an unexpectedly low flux (0–1.55% at slow growth rate and 0.02% at fast growth rate) through the later part of the TCA (from succinate to malate/oxolacetate) with the malate/oxaloacetate pool being replenished primarily using carbon fixing anaplerotic reactions. Another interesting feature of the BCG flux solutions obtained for both fast and slow growth rates was a negative flux (going in the reductive direction) through the α-ketoglutarate ferredoxin oxidoreductase (KOR) reaction. Approximately 11% of the oxalocetate originated from the anaplerotic PCK at slow growth rate, whereas at fast growth rate there was only a 1.2% anaplerotic flux through PCK with an additional 3.47% flux to malate/oxaloacetate through the reactions catalysed by either PCA or MEZ. Both slow growth rate solutions show a negative flux from succinate to succinyl CoA via succinyl-CoA synthetase (SCS) whereas at the fast growth rate there is negligible flux to succinyl CoA. Succinyl CoA is a precursor of methyl branched lipids, which are a major component of biomass, as well as being used for synthesis of diaminopepimelate, sulpholipids and heme. The increased flux to succinyl CoA may therefore reflect an increased requirement for some or all of these metabolites at slow growth rate. In contrast to the increased succinate/succinyl CoA output flux at slow growth rate, there was a dramatic increase in the output flux of acetyl CoA at the fast growth rate and this was also present in one of the two solutions for slow BCG cells.

### ^13^C MFA of *M. tuberculosis*

In order to confirm that the observed metabolic pathway for pyruvate dissimilation in slowly growing BCG was also occurring in *M. tuberculosis* we performed steady state chemostat cultivations of the tubercule bacillus at a dilution rate of 0.01 h^−1^ (t_d_ = 69.3 h). The steady state glycerol and Tween 80 consumption rates were very similar to those of BCG (Table 1). *M. tuberculosis* exhibited a lower CO_2_ production rate (0.23 mmol g biomass^−1^ h^−1^) compared to BCG (0.57 mmol g biomass^−1^ h^−1^).

**Figure 8:**
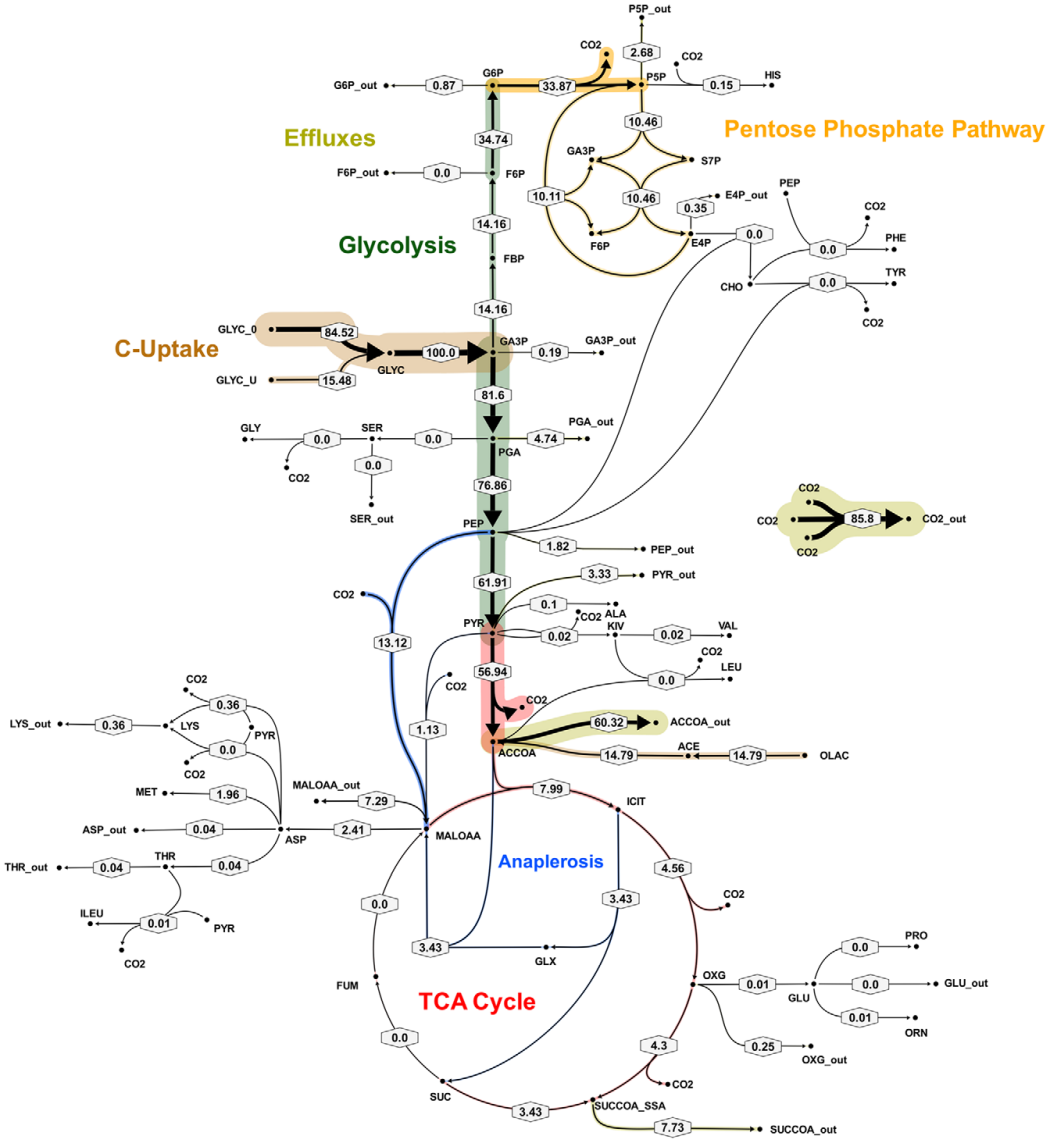
Metabolic flux map corresponding to solution A for *M. tuberculosis* in glycerol limited continuous culture at slow growth rate (t_d_ = 69 h). Flux values were normalized to the specific glycerol uptake rate which was arbitrarily given the value of 100. Both of the presented flux maps are able to describe the ^13^C labeling profiles of proteinogenic amino acids and measured extracellular flux values from steady state cultures of *M. tuberculosis* at slow growth rate. Arrows are pointing in the net flux direction and the width of each line is proportional to the underlying flux value. Numerical values of estimated fluxes (Table S5) are indicated on each flux arrow. The abbreviations are as in Figure 4.

**Figure 9:**
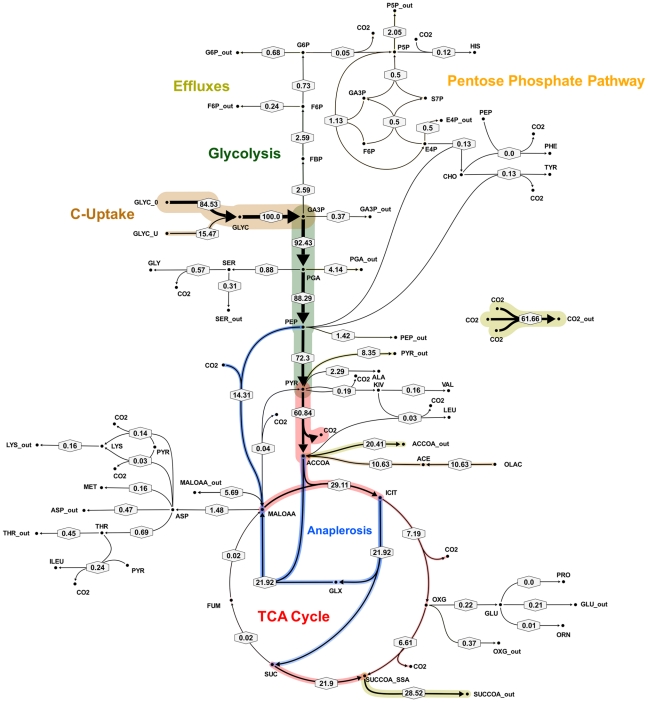
Metabolic flux map B for *M. tuberculosis* in glycerol limited continuous culture at slow growth rate (t_d_ = 69 h). Flux values were normalized to the specific glycerol uptake rate which was arbitrarily given the value of 100. Both of the presented flux maps are able to describe the ^13^C labeling profiles of proteinogenic amino acids and measured extracellular flux values from steady state cultures of *M. tuberculosis* at slow growth rate. Arrows are pointing in the net flux direction and the width of each line is proportional to the underlying flux value. Numerical values of estimated fluxes (Table S5) are indicated on each flux arrow. The abbreviations are as in Figure 4.

**Figure 10:**
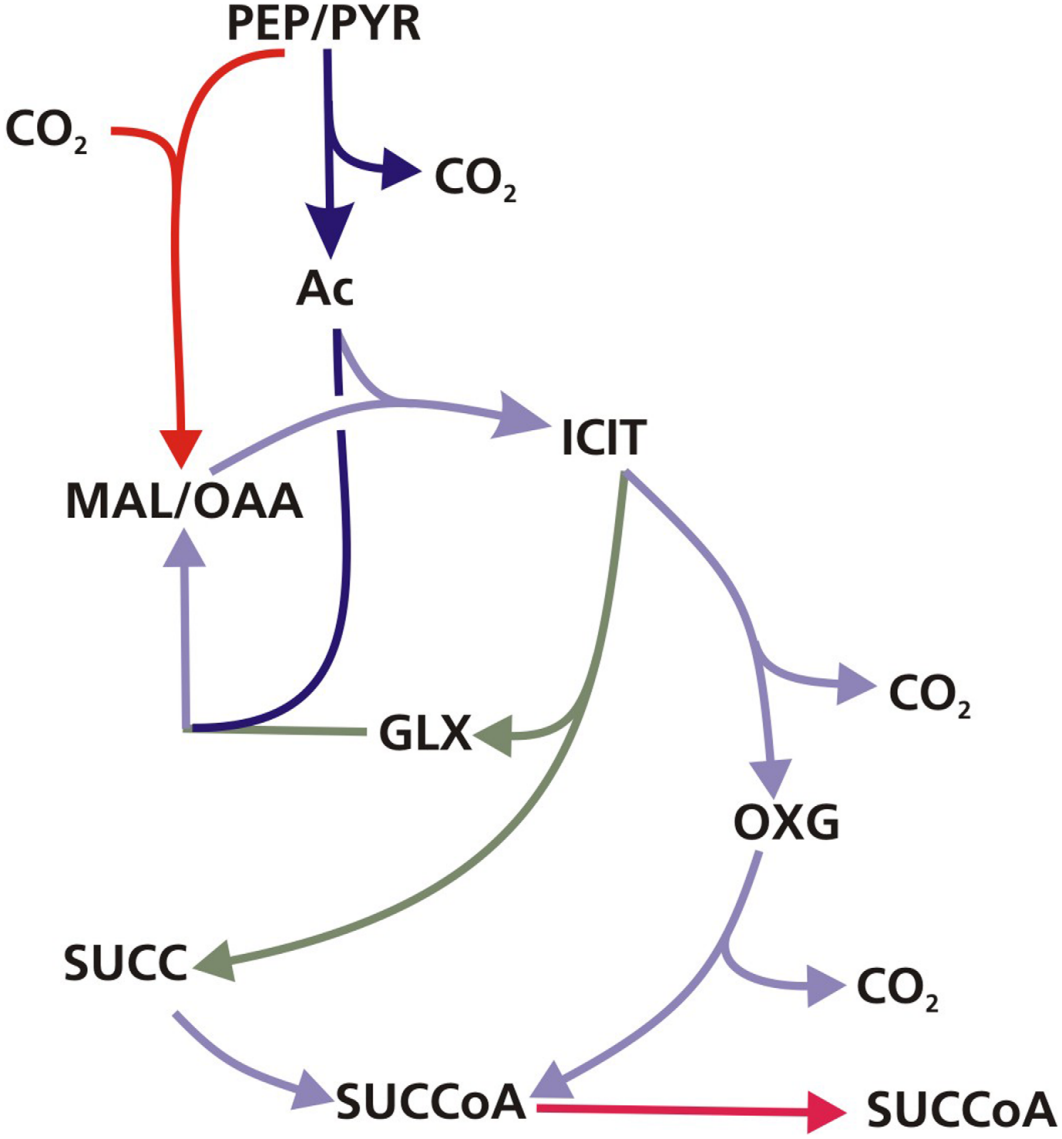
The GAS pathway. Schematic of the GAS pathway which is characterised by flux through the glyoxylate shunt and anaplerotic reactions for oxidation of pyruvate and succinyl CoA synthetase for the generation of succinyl CoA. The substrate abbreviations are as in Figure 4.

The metabolic fluxes for slowly growing *M. tuberculosis* were calculated from GC-MS data obtained from steady state [^13^C]glycerol labeling experiments by applying methodology similar to that used for BCG (Table S4). Again the flux values could not be determined unambiguously from the available labeling data and two best-fit solutions were obtained (Figure 8 and 9; Table S5). As found with BCG, the major differences between the two solutions were in the flux through gluconeogenesis, the pentose phosphate pathway and the output of acetyl CoA. Although the present data do not allow a unique flux solution, these data show that *M. tuberculosis* and BCG are operating the same route for pyruvate dissimilation which is characterized by flux through the glyoxylate shunt, synthesis of oxaloacetate primarily by an anaplerotic reaction from either phosphoenolpyruvate or pyruvate rather than via the TCA cycle and succinyl CoA synthetase for the generation of succinyl CoA . We have named this the GAS pathway (Figure 10) because of the usage of the **G**lyoxylate shunt, **A**naplerotic fixation of carbon from CO_2_ and **S**uccinyl CoA synthetase for the generation of succinyl CoA.

An interesting feature of the GAS pathway is the synthesis of oxaloacetate by anaplerotic reactions. These anaplerotic reactions involve assimilation of a carbon atom from CO_2_; so, to independently confirm this aspect of the flux solution we performed labeling experiments with [^13^C]sodium bicarbonate. Biomass samples were taken from steady state chemostat cultures of *Mycobacterium tuberculosis* growing at either fast or slow growth rate in media containing unlabeled glycerol and [^13^C]sodium bicarbonate. Because the rate of labeled and unlabeled CO_2_ uptake could not be accurately quantified (since bicarbonate is labile), it was not possible to perform full ^13^C-MFA analysis of these experiments. However, GC-MS analysis of the proteinogenic amino acids clearly demonstrated incorporation of the CO_2_-derived ^13^C into several amino acids including those derived from oxaloacetate, such as lysine (Table S4) at both the fast and slow growth rates, which is consistent with CO_2_ being fixed into biomass. Additionally, there were distinct differences in the degree of labeling of amino acids between fast and slow-growing cells. For instance, 22% of lysine is labeled with ^13^C at slow growth rate but 16% at fast growth rate. These ratios were reversed for methionine which is 16% labeled at fast growth rate but less than 5% at slow growth rate. These differences indicate growth rate-dependent shifts in the relative flux through anaplerotic carboxylating reactions.

## Discussion

The demonstration that ICL was essential for the multiplication of *M. tuberculosis* in macrophages and mice [5], [6] was interpreted as further evidence that fats are the major carbon source for this pathogen in the human host. Subsequent studies and the data presented here demonstrate that the function of ICL extends beyond lipid metabolism and that this enzyme has a role in the central metabolism of mycobacteria [2], [10], [11]. Our results show that ICL's were utilized even when glycerol was the major carbon source and that disruption of just one of the two functional *icl* genes impairs the viability of *M. bovis* BCG growing slowly in a carbon limited chemostat. Although the strain behaved like the parental strain at the faster growth rate (corresponding to a doubling time of 23 h), disruption of the *icl*1 gene resulted in a loss of viability at the slower growth rate (corresponding to a doubling time of 69 h). This finding echoes the previous demonstration that persistence of the *icl*1 mutant was attenuated in mice [5] and suggests that slow growth in the chemostat may simulate some aspects of *in vivo* growth. It also demonstrates the value of the chemostat for identifying growth-rate dependent phenotypes.

The essential requirement of *icl*1 for adaptation of BCG to slow growth under glycerol limiting conditions was not due to an increased consumption of the hydrolysis products of the detergent Tween 80 which is added to mycobacterial cultures to minimize clumping. Although some Tween 80 was metabolized by BCG during carbon limited growth in a chemostat, the Tween 80 consumption rate was significantly lower at slow growth rate than at the faster growth rate. In addition, the Tween 80 consumption rate did not differ significantly between the wild type and BCGΔ*icl*1. Whilst deletion of *icl*1did not reduce viability of *M. bovis* BCG at the faster growth rate in the chemostat, our findings indicate that the *icl*1 mutant had altered biomass yields and macromolecular composition, indicating that the *icl*1 gene is also playing a non-essential role in the physiology of BCG at the higher growth rate. Although it is unclear why the removal of *icl*1 results in an increased yield on glycerol it is consistent with similar findings for *Escherichia coli* where it was demonstrated that a functional *icl* gene was associated with a growth disadvantage in competition experiments in the chemostat [21]. The reduction in the carbohydrate content of the mutant cells indicate that the glyoxylate shunt may be involved in providing precursors for biosynthesis of non-essential carbohydrates. The metabolic cost of producing these carbohydrates is a possible explanation of the increased yield of the ICL mutant.

Using enzymatic methods and ^13^C-MFA we have identified a new route for pyruvate dissimilation which occurs in both BCG and *M. tuberculosis*, in which ICL is a key enzyme. The GAS pathway is characterized by flux through the glyoxylate shunt and also through the carbon fixing anaplerotic reactions at the PEP-pyruvate-oxaloacetate node (Figure 10). The very high flux through these ‘anaplerotic reactions’ are in excess of anaplerotic demands (Table S3) and therefore these reactions are also being used to oxidize pyruvate generated from glycolysis, in a similar manner to that described for the novel PEP-glyoxylate cycle in hungry *E. coli* [22], and to generate biomass. Another feature of the novel pathway is a very low flux through the succinate-oxaloacetate segment of the TCA cycle. Indeed it appears that most of the flux from the ICL reaction goes, not into the TCA cycle (to be used for catabolism), but into biosynthesis via succinyl CoA. Therefore under these conditions ICL plays an anabolic rather than a catabolic role (in fatty acid dissimilation). Given the importance of both ICL and PCK in the pathogenesis of the tubercle bacillus [6], [23] the discovery that it operates as part of this novel metabolic pathway is an important finding that may have implications for our understanding of how the tubercle bacillus survives *in vivo*. The fluxes through this novel pathway were significantly influenced by growth rate with a shift towards increased utilization of the glyoxylate shunt at slow growth rate accompanied by a diversion of precursors from synthesis of acetyl CoA to synthesis of succinate/succinyl CoA, which is consistent with our previous data indicating that the shift to slow growth rate is accompanied by changes in macromolecular composition [1]. Although two functioning ICL enzymes were identified in BCG, ICL2 has a significantly lower maximum activity [14] and, alone, may not be able to synthesize sufficient succinate/succinyl CoA for biosynthetic needs (e.g. synthesis of branched chain fatty acids), accounting for the observed essentiality of *icl*1 at slow growth rate. Indeed *icl*2 was unable to rescue an *icl M. smegmatis* mutant for growth on two carbon substrates or prevent the death of an *icl*1 mutant of *M. tuberculosis* during chronic TB infection in mice [5].

The ‘anaplerotic reactions’, PCK/PCA/MEZ, play a key role in the novel pathway we have described. These reactions may be used for gluconeogenesis but are also utilized to convert C_3_ acids to C_4_ acids. In *E. coli* this role is performed by phosphoenolpyruvate carboxylase but this enzyme is absent in *M. tuberculosis*. At the slow growth rate we found evidence that this function is performed primarily by PCK (operating in direction towards synthesis of oxaloacetate) in BCG and *M. tuberculosis*, as has been found for *Bacillus subtilis* [24]. This is interesting as PCK, like ICL is required for the growth of *M. tuberculosis* in macrophages and mice [23]. PCK catalyses the reversible conversion of oxaloacetate and phosphophoenolpyruvate and, with PCA and MEZ, controls the metabolic link between the TCA cycle and glycolysis represented by the PEP-pyruvate-oxaloacetate anaplerotic node. Previous data suggested that this enzyme predominantly functions in the gluconeogenic direction when *M. tuberculosis* is metabolizing glucose [23]. Here, we provide evidence that this enzyme can also operate anaplerotically during slow growth in a glycerol limited chemostat. Reversable fluxes from PEP to oxaloacetate would offer *M. tuberculosis* the metabolic flexibility to fine tune the balance of catabolic and replenishing reactions. This could be particularly important in the nutrient restricted human host.

At the fast growth rate, the flux solutions indicated that some carboxylating anaplerosis was catalysed by either PCA or MEZ. ^13^C-MFA was unable to distinguish which enzyme was responsible but we were unable to detect any activity of PCA *in vitro*. Although activity of this enzyme has been detected in *Mycobacterium smegmatis* [25] PCA activity has never been detected in either BCG or *M. tuberculosis*. However, the gene encoding this enzyme was identified as essential for growth of *M. tuberculosis* on media containing glycerol and glucose [26] and therefore we cannot rule out the possibility that the reaction conditions were not optimum for assaying this enzyme. The PCA of *Corynebacterium glutamicum* has been shown to be very unstable [27] and this may also be the case for BCG. Malic enzyme decarboxylates malate to pyruvate and is thought to be important in providing acetate and NADPH for the biosynthesis of lipids. Although this enzyme can function anaplerotically, in other bacteria it is generally thought to operate in the gluconeogenic direction. Low levels of MEZ activity was detected in the BCG cells but this activity was unaffected by growth rate. Recent ^13^C data has provided evidence for malic enzyme activity in *M. tuberculosis* [23] whereas early studies had suggested that activity was very low or absent in slow growing mycobacteria [17]. Further studies are required to elucidate precisely which enzymes are involved in these important reactions.

Isotopic tracer experiments using sodium [^13^C]bicarbonate confirmed that, in accordance with the flux solutions, an appreciable proportion (up to 22% incorporation in some amino acids after the addition of one volume of media containing sodium [^13^C]bicarbonate) of carbon in *M. tuberculosis* growing in a glycerol limited chemostat arises from CO_2_ fixation through carboxylation reactions. The estimated flux through carboxylating anaplerotic reactions is much more than the relatively modest requirement for anaplerotic replenishment of the TCA cycle intermediates due to their removal for amino acid synthesis (less than 1% of total flux) and suggests that fixed CO_2_ is being incorporated into other components of biomass, as well as amino acids. These data alongside a number of different studies provide evidence that CO_2_ fixation maybe important for the growth and virulence of *M. tuberculosis*. The stimulatory effect of CO_2_ on the growth of mycobacterium has been known for decades [28] and also ^14^C tracer experiments performed in the 1950's demonstrated that *M. tuberculosis* could use CO_2_ as a source of carbon [29], [30]. It has also been shown that physiological relevant levels of CO_2_ prevent the growth arrest of *M. bovis* BCG in a hypoxic model of persistence [31]. In addition, *M. tuberculosis* encodes three carbonic anhydrases, Rv1284, Rv3273 and Rv3588c which have a role in the growth and virulence of *M. tuberculosis*: Rv3588c is essential for growth *in vivo* [32] and Rv1284 is essential for *in vitro* growth [26] and was also highly upregulated in a starvation model of persistence [33]. These enzymes catalyse the reversible hydration of CO_2_ to bicarbonate and are associated with metabolic enzymes such as PCK, PCA and MEZ which produce or consume CO_2_ or bicarbonate.

It is unlikely that *M. tuberculosis* is operating either the Calvin, Wood-Ljungdahl or the 3-hydroxypropionate cycles to fix CO_2_ as this pathogen does not have a ribulose-bisphosphate carboxylase (RuBisCO) [34] and the genes for malyl CoA lyase or formate-tetrahydrofolate ligase are not annotated in the genome [35]. The presence of KOR and a gene homologous to the ATP-citrate lyase ß-chain (*cit*E, Rv2498c) has led to suggestions that a reductive tricarboxylic acid cycle may function to fix CO_2_ in this bacterium [34], [36]. The flux solutions presented here do indeed suggest that at least the KOR reaction may operate in the reductive (carboxylating) direction in some circumstances. Operation of the entire reductive TCA pathway seems unlikely as *M. tuberculosis* lacks the α and γ-subunits of citrate lyase required for the reductive TCA and studies suggest that CitE functions in fatty acid biosynthesis [37]. The pattern of incorporation of bicarbonate-derived ^13^C differed between fast and slow growth rate, with lysine being the predominant labeled product at slow growth rate but methionine being the predominant labeled product at fast growth rate. The carbon backbone of both amino acids is derived from oxaloacetate supporting anaplerotic flux via PCK as being the predominant carboxylating reaction at both growth rates. However, as both amino acids do share the same four carbon backbone, the shift in label accumulation between lysine and methionine at slow and fast growth rate is puzzling. One possibility is that the difference is due to differences in the degree of labeling of the methyl group of methionine, which is not derived from oxaloacetate but via a methylation reaction performed by methionine synthase with methylated tetrahydrofolate being the C1 source. The ultimate source of the carbon atom in C1 metabolism of mycobacteria has however not been determined.

### Conclusions

This study is the first to use ^13^C-MFA to investigate the metabolism of a mycobacterium. Using this method we identify a novel metabolic pathway operating in *M. tuberculosis* which we have named the GAS pathway (Figure 8) because it utilizes the glyoxylate shunt and anaplerotic reactions for oxidation of pyruvate combined with very low flux through the succinate – oxaloacetate segment of the TCA cycle. These results also add to a growing body of evidence which indicates that ICL is involved in processes beyond lipid metabolism *in vitro*, and probably also *in vivo*. We demonstrate that the fluxes through this novel pathway are influenced by growth rate such that one of the two ICL's becomes essential at slow growth rate. This essentiality is unrelated to lipid catabolism and appears to reflect an anabolic shift at slow growth rate, possibly towards synthesis of complex lipids. The relevance of these findings to the *in vivo* situation is demonstrated by our previous finding that adaptation *M. tuberculosis* to growth in macrophages also involves a shift to slow growth. The GAS pathway is also characterized by significant levels of CO_2_ fixation through anaplerotic reactions that generate oxaloacetate. We were able to confirm significant levels of CO_2_ fixation in *M. tuberculosis*. Given the importance of the glyoxylate shunt in the pathogenesis of the tubercule bacillus and the abundance of CO_2_ in the human host, the discovery of this novel pathway is an important finding that is likely to be relevant to the pathogenesis of tuberculosis.

## Materials and Methods

### Bacterial strains and growth conditions

*M. bovis* BCG strain (ATCC 35748) or *Mycobacterium tuberculosis* (H37Rv) was cultured in a 2- l bioreactor (Adaptive Biosystem Voyager) under aerobic conditions and at pH 6.6 as previously described [1]. Chemostat cultures were grown in Roisin's minimal medium at a constant dilution rate of 0.03 h^−1^ (equivalent to a doubling time, t_d_ of 23 h) or 0.01 h^−1^ (t_d_ = 69 h). Culture samples were withdrawn from the chemostat to monitor cellular dry weight, viable counts, optical density, nutrient utilization and CO_2_ and O_2_ levels were measured in the exhaust gas [1]. After waiting five residence times at a particular dilution rate, the reactor was assumed to be at steady state if the OD, glycerol concentration, oxygen uptake rate and CO_2_ production rate were steady within a 15% range for 48 h. Once the steady state was reached cells were harvested for analysis.

The ^13^C labeling experiments were initiated after the chemostat culture was in steady state by replacing the feed medium with an identical medium containing a mixture of 1 ml [^13^C_3_]glycerol and 4 ml of natural glycerol l^−1^. For the carbon fixation chemostat experiments a modified Roisin's medium was used which contained 0.15% sodium bicarbonate [38]. After steady state was achieved the medium was replaced with an identical medium containing 0.15% sodium [^13^C]bicarbonate. Since CO_2_ is rapidly lost to air from a stirred solution of sodium bicarbonate, the chemostat air supply was made recyclable by connecting the effluent gas outlet to the air inlet. Oxygen concentrations were monitored carefully to ensure that oxygen levels remained above 70%. Biomass samples for GC-MS analysis were taken for analysis every volume change.

### Culture analysis

Biomass and supernatant samples were collected and harvested [1]. Biomass was determined according to the method described by Lynch and Bushell [39]. The amounts of glycerol in the supernatant and in fresh medium were assayed by use of a commercial assay kit that employs a glycerokinase-coupled enzyme assay system (Boehringer Mannheim). To assay for Tween 80 supernatant samples were hydrolysed by boiling for 1 h in methanolic KOH (5% potassium hydroxide: 50% methanol). After neutralizing the samples with HCl the free fatty acids were assayed using a commercial kit (Roche). Non-hydrolysed samples were also analyzed to measure any free fatty acids present in the supernatant.

### Macromolecular composition

The macromolecular composition of the cellular biomass was determined from freeze-dried cell pellets as previously described [1].

### Strain construction

For the construction of an isocitrate lyase mutant, *E. coli* strain DH5α was grown in solid or liquid Luria-Bertani (LB) medium as described by Sambrook *et al*. [40]. *M. bovis* BCG cells were propagated in Middlebrook 7H9 broth or 7H11 agar containing 5% (v/v) OADC enrichment media supplement (Becton Dickenson), 0.5% glycerol plus 0.05% Tween 80 for liquid cultures. For mycobacteria, when selection was required, kanamycin at 20 µg ml^−1^, X-gal at 50 µg ml^−1^ and sucrose at 2% (w/v) were added to the culture media.

An unmarked isocitrate lyase mutant of *M. bovis* BCG was constructed using the strategy described by Parish and Stoker [41]. PCR was used to amplify two separate 1 kb DNA regions flanking the *M. bovis* BCG *icl*1 gene. These fragments were cloned into p2NIL to generate p2NIL-*icl*1. The final delivery vector was generated by cloning the *Pac* I cassette from pGOAL19 into the p2NIL-*icl*1 construct to give pUS*icl*1. Single crossovers were selected for on 7H11 agar supplemented with kanamycin and X-gal and then grown in media lacking antibiotics to allow the second recombination to occur. Following growth the cells were sub-cultured onto sucrose plates to select for sucrose-resistant white colonies, which had lost the integrated plasmid through a second crossover. PCR and Southern analysis were performed to verify the expected genotypes.

### Analysis of *in vitro* enzyme activities

Crude enzyme extracts were prepared according to Tian *et al* (2005) with minor modifications [2]. Protein was assayed using the Bio-Rad DC protein assay kit and following the manufacturer's instructions. All reaction mixtures (1 ml) contained 100 µg of *M. bovis* BCG lysates.

#### Isocitrate dehydrogenase (ICDH)

ICDH was assayed as described [19].

#### Phosphoenolpyruvate carboxykinase (PCK)

The procedure described by Mukhopadhyay *et al* (2001) was used for assaying PCK activity in the direction of oxaloacetate formation [42].

#### Malic enzyme (malate dehydogenase, decarboxylating) (MEZ)

A modification of previously described procedures was used to assay for MEZ activity [17], [43], [44]. The reaction mixture contained 100 mM Tris-HCl (pH 7.8), 5 mM MnCl_2_, 40 mM sodium l-malate, 0.6 mM NADP^+^, 20 mM KCl. Reactions were monitored spectrophotometrically by following the production of NADPH at 340 nm (ε = 6.223 M^−1^ cm^−1^) at 30°C.

#### Pyruvate carboxylase (PCA)

PCA was assayed exactly as described [25].

#### Glycine dehydrogenase (GDH)

GDH was monitored by the method described by Wayne (1996) [45].

### Construction of an isotopomer model of central metabolism in *M. tuberculosis*

An isotopomer model of central metabolism (Figure 3, Table S1) in *M. tuberculosis* and *M. bovis* BCG was constructed in the framework of 13CFlux [18] (http://www.13cflux.net/), a software toolbox for flux distribution estimation.

The model was based on our GSMN-TB genome-scale model [2] of *M. tuberculosis* and includes reactions of glycolysis (EMP), the pentose phosphate pathway (PPP), the tricarboxylic acid cycle (TCA) and anaplerosis (ANA). Moreover, all biosynthesis pathways relevant for *M. tuberculosis* are formulated in simplified form in that linear reaction sequences are combined into one reaction (Figure S1). The scrambling reaction tca6a/b is assumed to proceed in equal proportion. This metabolic network model was supplemented with carbon atom transitions and consisted of a total of 75 reactions. Flux values for net and exchange rates can be derived from 47 independent flux parameters (35 net +12 exchange) that are estimated using 137 labeling measurements (mass isotopomers of amino acids, Table S3) and 30 flux measurements obtained from biomass hydrolysates or calculated from intermediates (25 precursor requirements, (Table S4), (labeled) glycerol and Tween 80 uptake rates (Table 1) as well as the overall CO_2_ net production rate.

In order to incorporate enzyme activities, steady state data for experiments with different growth rates *µ_S_* = 0.01 h^−1^ and *µ_F_* = 0.03 h^−1^ are fitted in one model. This is achieved by doubling all reaction equations of the basic flux model (Figure S1). The two sub-models are then “loosely” connected via inequality constraints reflecting the tendencies of measured activities between slow and fast growth for the enzymes PCK, ICL and ICDH (see also results).

### Biomass hydrolysate and preparation of amino acid derivatives

Biomass samples from the ^13^C labeled chemostat cultures were washed and hydrolysed as described by Borodina *et al*, 2008 [46]. The dried hydrosylate was dissolved in 1 ml norvaline solution (0.075 mM in 80∶20 H_2_O:MeOH). 0.1 ml was dried *in-vacuo* and this mixture was derivatised by adding 140 µl acetonitrile:N-tert-butyldimethylsilyl-N-methyltrifluoroacetamide (MTBSTFA):1% tert-butyldimethylchlorosilane (TBDMCSI), 1:1., sonicating (room temperature, 30 min) and then heating (90°C, 30 min) to complete the derivatisation. Samples were analysed by Gas Chromatography-Mass Spectrometry (GC-MS) within 72 h after derivitisation. The concentration of protein in each hydrolysate was determined using the Lowry method [47]. Bovine serum albumin was used as a standard and was included with each set of samples.

### GC-MS analysis

The analysis was performed with a 6890N Network GC system (Agilent) fitted with a DB-5ms capillary column (15 m×0.18 mm internal diameter×0.18 µm film with 5 m Duraguard integrated guard column) and deactivated quartz wool packed FocusLiner (SGE) coupled to a Pegasus III time-of-flight (TOF) mass spectrometer (Leco) equipped with a splitless injector. The injector temperature was initially held at 70°C for 2 min followed by heating to 350°C at a rate of 17°C min^−1^. This temperature was held for 1.5 min. The flow was held constant at 1.4 ml He min^−1^. Injection volume, 5 µl. Inlet temperature, 250°C. Interface temperature, 310°C. Source temperature, 245°C. The system was operated with a mass range of 40–800 m/z at an EM voltage of 70 V with a spectral acquisition rate of 20 spectra s^−1^.

^13^C isotopologue abundances (i.e., ^13^C incorporation; ^12^Cn, ^13^C1, …, ^13^Cn) for each amino acid were determined for fragments containing the intact carbon skeleton for each amino acid; generally using the [M-57]^+^ ion. To obtain further information relating to the ^13^C incorporation at individual positions within individual amino acids, isotopologue abundances were also determined for fragments formed by cleavage of the C1–C2 bond in some samples. Within a subset of these, isotopologue abundances for the fragment formed by cleavage of the C2–C3 bond in serine were also determined allowing comprehensive characterization of the isotopic incorporation into serine. Mass spectra of the derivatized amino acids were corrected for the natural abundance of all stable isotopes.

### Flux parameter estimation

Non-linear weighted least squares fitting is applied in order to determine the flux values which are the most likely description of the labeling data and biomass constraints [48]. Initial flux distributions are generated by Monte Carlo sampling and used for a multi-start optimization. For validation of the solution, flux or labeling measurements (whole groups, C2-Cn fragments or single measurements) that fit the least are combinatorially removed from the data set. Solutions obtained using the reduced data set that are consistent with the best fit gives an indication that the estimated flux values describe the experimental data set.

### Statistical analysis

Based on normally distributed measurement errors, flux errors were calculated using a linearized statistical approach [48]. Phosphoglucose isomerise (PGI) and PCK were constrained to fitted values in order to determine confidence levels for the split ratio between glycolysis and the pentose phosphate pathway as well as the reaction cycle consisting of PCK, PCA/MEZ, pyruvate kinase (PK). These fluxes are comparably low and and so this was not a severe restriction. As is common practice all net fluxes in direction of biomass synthesis are likewise constrained. Resulting flux standard deviations are given in Table S4.

## Supporting Information

Figure S1 **Southern analysis of the** ***icl*** **1 mutant.** Chromosomal DNA isolated from WT *M. bovis* BCG (lane 2) and Δicl (lane 3) were digested with *Bgl*II and transferred to a nylon membrane. The membrane was probed with the digoxigenin labeled probe HR containing the *icl*1 gene and its flanking regions. The WT fragments are the expected 3.99 kb and 1.9 kb fragments while the genomic DNA from the Δ*icl* had the expected 2.62 kb fragment. The sizes of the bands were determined from the migration distance of the DNA molecular mass marker *EcoR*I SPP1 (Roche ladder number VII) which is present in lane 1.
(TIF)

Table S1 **Non stationary and stationary ^13^C isotopomer abundance of amino acids from protein hydrolysis.** Incorporation of ^13^C label into the protein amino acids of *M. bovis* BCG cells grown in a chemostat at a dilution rate of 0.01 h^−1^ with 20% [^13^C_3_]glycerol.
(XLS)

Table S2 **Network model of the central metabolism of** ***Mycobacterium bovis*** **BCG and** ***Mycobacterium tuberculosi*** **s.** Stoichiometry and carbon transformations for reactions in the network of *M. bovis* BCG and *M. tuberculosis*. This network consists of 75 reactions.
(DOC)

Table S3 **Experimentally derived requirements and estimated effluxes from intermediate and amino acid pools for biomass synthesis of** ***Mycobacterium bovis*** **BCG (BCG) and** ***Mycobacterium tuberculosis*** **(MTB) growing in a chemostat.** The precursor requirements for amino acid pools were derived from measurements of the macromolecular composition of biomass hydrolysates.
(XLS)

Table S4 **Experimental (GC-MS) and calculated mass distributions in the amino acid derivatives of** ***Mycobacterium bovis*** **BCG (BCG) and** ***Mycobacterium tuberculosis*** **(MTB) growing in a steady state chemostat.** GC-MS measured values for the ^13^C incorporation into proteogenic amino acids as compared with the values calculated using 13CFlux.
(XLS)

Table S5 **Estimated metabolic fluxes of** ***Mycobacterium bovis*** **BCG (BCG) and** ***Mycobacterium tuberculosis*** **(MTB).** *In vivo* fluxes for glycerol limited *M. bovis* BCG and *M. tuberculosis* growing at either fast (t_d_ = 23 h) or a slow (t_d_ = 69 h) growth rate in a chemostat.
(XLS)

Table S6 **Abbreviations of metabolite names.** Explicit names of enzymes and metabolites used in this work.
(DOC)
