# Supplementary figures and images for: 13C Metabolic Flux Analysis Identifies an Unusual Route for Pyruvate Dissimilation in Mycobacteria which Requires Isocitrate Lyase and Carbon Dioxide Fixation
Source: PLoS Pathog. 2011 Jul 21;7(7):e1002091. doi: 10.1371/journal.ppat.1002091 (PMC3141028; doi:10.1371/journal.ppat.1002091)

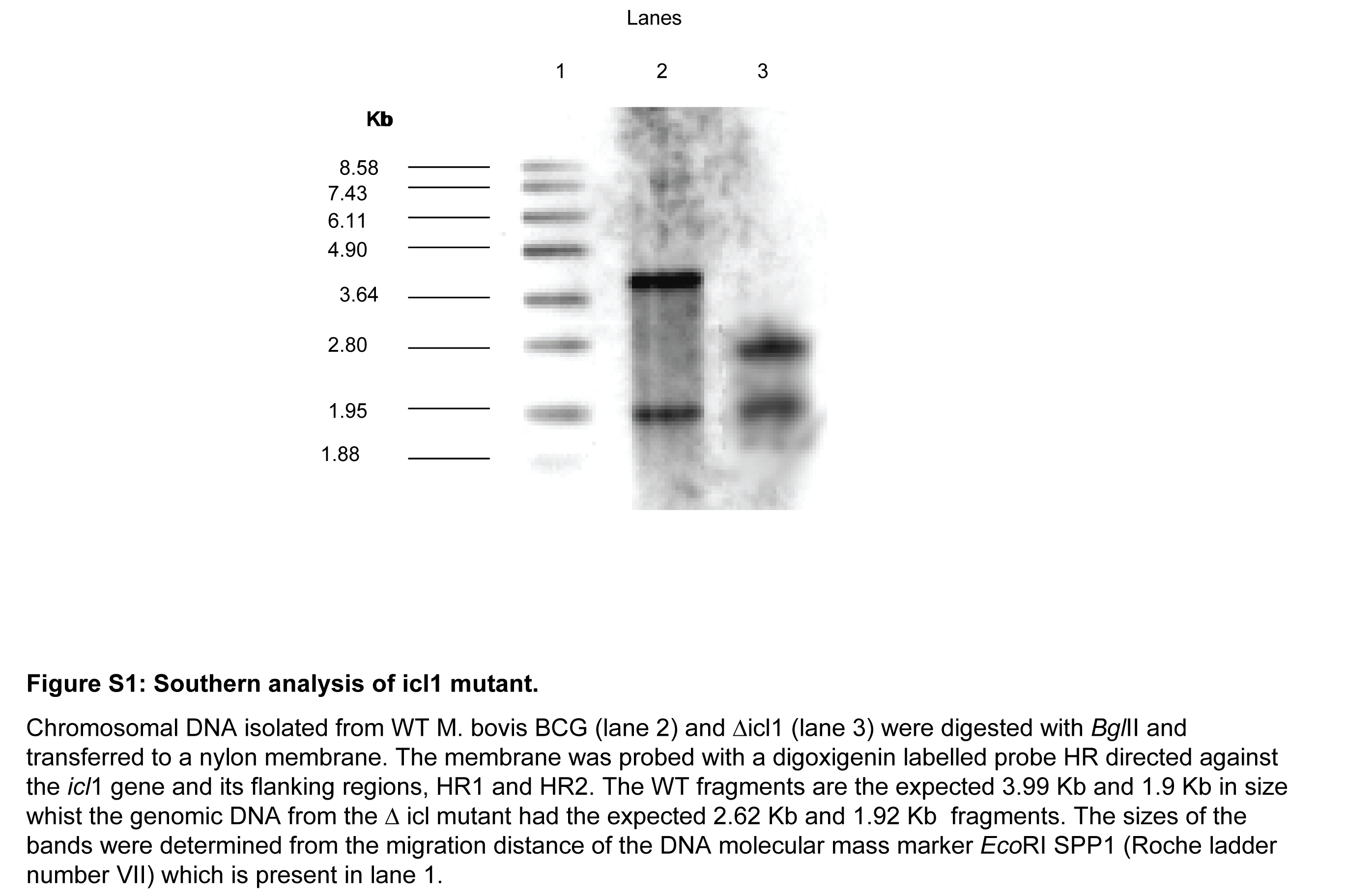

Supplement: Figure S1 — Southern analysis of the icl 1 mutant. Chromosomal DNA isolated from WT M. bovis BCG (lane 2) and Δicl (lane 3) were digested with BglII and transferred to a nylon membrane. The membrane was probed with the digoxigenin labeled probe HR containing the icl1 gene and its flanking regions. The WT fragments are the expected 3.99 kb and 1.9 kb fragments while the genomic DNA from the Δicl had the expected 2.62 kb fragment. The sizes of the bands were determined from the migration distance of the DNA molecular mass marker EcoRI SPP1 (Roche ladder number VII) which is present in lane 1. (TIF) [file ppat.1002091.s001.tif]
