# Supplementary material for: 13C Metabolic Flux Analysis Identifies an Unusual Route for Pyruvate Dissimilation in Mycobacteria which Requires Isocitrate Lyase and Carbon Dioxide Fixation
Source: PLoS Pathog. 2011 Jul 21;7(7):e1002091. doi: 10.1371/journal.ppat.1002091 (PMC3141028; doi:10.1371/journal.ppat.1002091)
Supplement: Table S2 — Network model of the central metabolism of Mycobacterium bovis BCG and Mycobacterium tuberculosi s. Stoichiometry and carbon transformations for reactions in the network of M. bovis BCG and M. tuberculosis. This network consists of 75 reactions. (DOC) [file ppat.1002091.s003.doc]

Table S2: Network model of the central metabolism of *Mycobacterium bovis* BCG and *Mycobacterium tuberculosis*

Linear reaction sequences were condensed for simplification. Irreversibility assumptions are derived from thermodynamic considerations and represented by corresponding arrow directions.

| **Reaction** | **Stoichiometry and C-atom transitions** |
| --- | --- |
| GLYC_upt_ | GLYC (#ABC) → GA3P (#ABC) |
| OLAC_upt_ | OLAC (#AB) → ACE (#AB) |
| CO_2out_ | CO_2_ (#A) → CO_2ex_ (#AB) |
| SUCCOA_out_ | SUCCOA (#ABCD) → SUCCOA_ex_ (#ABCD) |
| pgi | F6P (#ABCDEF) ↔ G6P (#ABCDEF) |
| fbp | FBP (#ABCDEF) ↔ F6P (#ABCDEF) |
| fba | GA3P (#CBA) + GA3P (#DEF) ↔ FBP (#ABCDEF) |
| gabA | GA3P (#ABC) ↔ PGA (#ABC) |
| eno | PGA (#ABC) ↔ PEP (#ABC) |
| pyk | PEP (#ABC) ↔ PYR (#ABC) |
| gnd | G6P (#ABCDEF) → P5P (#BCDEF) + CO_2_ (#A) |
| tkt_1_ | GA3P (#CDE) + F6P (#ABabcd) ↔ P5P (#ABCDE) + E4P (#abcd) |
| tkt_2_ | S7P (#ABabcde) + GA3P (#CDE) ↔ P5P (#ABCDE) + P5P (#abcde) |
| tal | E4P (#defg) + F6P (#abcABC) ↔ GA3P (#ABC) + S7P (#abcdefg) |
| pdh | PYR (#ABC) → ACCOA (#BC) + CO_2_ (#A) |
| cs | MALOAA (#ABCD) + ACCOA (#ab) → ICIT (#DCBbaA) |
| icdh | ICIT (#ABCDEF) ↔ OXG (#ABCDE) + CO_2_ (#F) |
| kor  kgd* | OXG (#ABCDE) ↔ SUCCOA (#BCDE) + CO_2_ (#A)  OXG (#ABCDE)  SSA (#BCDE) + CO_2_ (#A) |
| scs  gabD1/D2* | SUCCOA (#ABCD) ↔ SUC (#ABCD)  SSA (#ABCD)  SUC (#ABCD) |
| sdh | SUC (#ABCD) ↔ FUM (#ABCD) |
| fum_a/b_ | FUM (#ABCD) ↔ MALOAA (0.5 #ABCD + 0.5 #DCBA) |
| icl | ICIT (#ABCDEF) → GLX (#AB) + SUC (#FCDE) |
| ms | GLX (#AB) + ACCOA (#ab) → MALOAA (#ABba) |
| pck | PEP (#ABC) + CO_2_ (#a) ↔ MALOAA (#ABCa) |
| mez | PYR (#ABC) + CO_2_ (#D) ↔ MALOAA (#ABCD) |
| ALA_bs_ | PYR (#ABC) → ALA (#ABC) |
| VAL_bs1_ | PYR (#ABC) + PYR (#abc) → KIV (#ABCbc) + CO_2_ (#a) |
| VAL_bs2_ | KIV (#ABCD) → VAL (#ABCD) |
| LEU_bs_ | KIV (#ABCDE) + ACCOA (#ab) → LEU (#BCDEab) + CO_2_ (#A) |
| SER_bs_ | GA3P (#ABC) → SER (#ABC) |
| GLY_bs_ | SER (#ABC) → GLY (#AB) + CO_2_ (#C) |
| HIS_bs_ | P5P (#ABCDE) → HIS (#EDCBA) |
| ASP_bs_ | MALOAA (#ABCD) → ASP (#ABCD) |
| THR_bs_ | ASP (#ABCD) → THR (#ABCD) |
| MET_bs_ | ASP (#ABCD) → MET (#ABCD) |
| LYS_bs_ | PYR (#ABC) + ASP (#ABCD) → LYS (0.5 #BCabcd + 0.5 #ABCbcd) + CO_2_ (0.5 #A + 0.5 #a) |
| ILEU_bs_ | THR (#ABCD) + PYR (#abc) → ILEU (#ABCDbc) + CO_2_ (#a) |
| GLU_bs_ | OXG (#ABCDE) → GLU (#ABCDE) |
| PRO_bs_ | GLU (#ABCDE) → PRO (#ABCDE) |
| ORN_bs_ | GLU (#ABCDE) → ORN (#ABCDE) |
| PHE_bs1_ | E4P (#ABCD) + PEP (#abc) → CHO (#bcABCDa) |
| PHE_bs2_ | CHO (#ABCDEFG) + PEP (#abc) → PHE (#abcABCDEF) + CO_2_ (#G) |
| TYR_bs_ | CHO (#ABCDEFG) + PEP (#abc) → TYR (#abcABCDEF) + CO_2_ (#G) |

*The TCA pathways kor-scs and kgd-gabD1/D2 are operating in parallel and cannot be distinguished by 13C flux and therefore the pools SUCCOA and SSA are lumped (SUCCOA_SSA).
