# Supplementary material for: 13C Metabolic Flux Analysis Identifies an Unusual Route for Pyruvate Dissimilation in Mycobacteria which Requires Isocitrate Lyase and Carbon Dioxide Fixation
Source: PLoS Pathog. 2011 Jul 21;7(7):e1002091. doi: 10.1371/journal.ppat.1002091 (PMC3141028; doi:10.1371/journal.ppat.1002091)
Supplement: Table S6 — Abbreviations of metabolite names. Explicit names of enzymes and metabolites used in this work. (DOC) [file ppat.1002091.s007.doc]

Table S6. Abbreviations of metabolite names.

| **Abbrev.** | **metabolite** | **KEGG ID** |
| --- | --- | --- |
| ACCOA | Acetyl-CoA | C00024 |
| ACE | Acetate | C00033 |
| ALA | L-Alanine | C00041 |
| ASP | L-Aspartate | C00049 |
| CHO | Chorismate | C00251 |
| CO_2_ | CO2 | C00011 |
| E4P | D-Erythrose 4-phosphate | C00279 |
| F6P | D-Fructose 6-phosphate | C00085 |
| FBP | D-Fructose 1,6-bisphosphate | C00354 |
| FUM | Fumarate | C00122 |
| G6P | D-Glucose 6-phosphate | C00092 |
| GA3P | Glyceraldehyde 3-phosphate | C00661 |
| GLU | L-Glutamate | C00025 |
| GLX | Glyoxylate | C00048 |
| GLY | Glycine | C00037 |
| GLYC | Glycerol | C00116 |
| HIS | L-Histidine | C00135 |
| ICIT | Isocitrate | C00311 |
|  | Citrate | C00158 |
| ILEU | L-Isoleucine | C00407 |
| KIV | 2-Oxoisovalerate | C00141 |
| LEU | L-Leucine | C00123 |
| LYS | L-Lysine | C00047 |
| MALOAA | L-Malate | C00149 |
|  | Oxaloacetate | C00036 |
| MET | L-Methionine | C00073 |
| OLAC | Oleic acid | C00712 |
| ORN | Ornithine | C01602 |
| OXG | 2-Oxoglutarate | C00026 |
| P5P | alpha-D-Ribose 5-phosphate (R5P) | C00117 |
|  | L-Ribulose 5-phosphate | C01101 |
|  | L-Xylulose 5-phosphate | C03291 |
| PEP | Phosphoenolpyruvate | C00074 |
| PGA | 2-Phospho-D-glycerate | C00631 |
|  | 3-Phospho-D-glycerate | C00197 |
| PHE | L-Phenylalanine | C00079 |
| PRO | L-Proline | C00148 |
| PYR | Pyruvate | C00022 |
| S7P | Sedoheptulose 7-phosphate | C00281 |
| SER | L-Serine | C00065 |
| SUC | Succinate | C00042 |
| SUCCOA | Succinyl-CoA | C00091 |
| THR | L-Threonine | C00188 |
| TRP | L-Tryptophan | C00078 |
| TYR | L-Tyrosine | C00082 |
| VAL | L-Valine | C00183 |
